# The Influence of Bilectalism and Non-standardization on the Perception of Native Grammatical Variants

**DOI:** 10.3389/fpsyg.2017.00205

**Published:** 2017-02-20

**Authors:** Evelina Leivada, Elena Papadopoulou, Maria Kambanaros, Kleanthes K. Grohmann

**Affiliations:** ^1^Department of Rehabilitation Sciences, Cyprus University of TechnologyLimassol, Cyprus; ^2^Cyprus Acquisition Team, University of CyprusNicosia, Cyprus; ^3^Department of Humanities, European University CyprusNicosia, Cyprus; ^4^Department of English Studies, University of CyprusNicosia, Cyprus

**Keywords:** bilectalism, dialect, grammatical variants, non-standardization

## Abstract

Research in speakers of closely related varieties has shown that bilectalism and non-standardization affect speakers’ perception of the variants that exist in their native languages in a way that is absent from the performance of their monolingual peers. One possible explanation for this difference is that non-standardization blurs the boundaries of grammatical variants and increases grammatical fluidity. Affected by such factors, bilectals become less accurate in identifying the variety to which a grammatical variant pertains. Another explanation is that their differential performance derives from the fact that they are competent in two varieties. Under this scenario, the difference is due to the existence of two linguistic systems in the course of development, and not to how close or standardized these systems are. This study employs a novel variety-judgment task in order to elucidate which of the two explanations holds. Having administered the task to monolinguals, bilectals, and bilinguals, including heritage language learners and L1 attriters, we obtained a dataset of 16,245 sentences. The analysis shows differential performance between bilectal and bilingual speakers, granting support for the first explanation. We discuss the role of factors such as non-standardization and linguistic proximity in language development and flesh out the implications of the results in relation to different developmental trajectories.

## Introduction

Linguistic research has shown that non-standard varieties allow for greater grammatical fluidity in a way that blurs the boundaries across them and affects speakers’ perception of whether a specific variant belongs to their linguistic repertoire or not (Cheshire and Stein, 1997; Henry, 2005). Non-standardization affects not only cross-linguistic boundaries but also the norms of acceptability that define variants (Milroy, 2001). This, in turn, affects speakers’ perception and ultimate performance of grammatical variants in their native variety or varieties (Henry, 2005; Papadopoulou et al., 2014). A second important factor that affects linguistic development is variation in the input, as happens when the linguistic environment involves exposure to more than one language. Bilingual speakers benefit from the cognitive advantages of bilingualism, which have an impact on the processing mechanisms that are active during the acquisition process. For example, bilingualism strengthens the development of the attentional control abilities with the effect persisting throughout the lifetime (Bialystok et al., 2004; Laka, 2012). Linguistic proximity across the different languages a child is exposed to is a third key factor that affects bi- or multilingual development and cross-linguistic transfer (Grohmann, 2014; Garraffa et al., 2015; Westergaard et al., 2016).

The interaction of these three factors ultimately invests the linguistic development of speakers that become exposed to two *closely related varieties*—henceforth, ‘(discrete) bilectal’ speakers (Rowe and Grohmann, 2013)—with a cluster of unique properties that can be described as inherent to the notion of ‘dialect design’ (Grohmann and Leivada, 2012): (i) blurred boundaries of grammatical variants, (ii) dialect continua and the emergence of intermediate speech repertoires (Cornips, 2006), possible lack of codification, and a prescriptive notion of correctness that interferes with speakers’ perception of their own linguistic repertoire (Henry, 2005; Grohmann and Leivada, 2012).

A striking result from the field of experimental linguistics relates to the finding that a native speaker may judge a certain variant or form to be completely unacceptable, but be recorded producing it in their own speech (Cornips and Poletto, 2005). If this is true in cases of mono- or bilingual development, in bilectal development it involves non-official/-codified—and as such, more fluid—varieties, rendering an even greater degree of discrepancy between speakers’ introspective judgments about their repertoire and the actual linguistic repertoire itself. Even in the absence of closely related varieties and the dialect design, bilingualism may leave its imprint on speakers’ performance in acceptability judgment tasks.^1^ This results in observing differential performance across mono- and bilingual populations in both off-line measures (e.g., the higher acceptance rate of over-regularizations in bilinguals reported in Jacobson and Cairns, 2008) and on-line measures (e.g., the slower reaction times of balanced bilinguals in Foursha et al., 2006). Experiments measuring acceptability judgment using event-related potentials have also shown lower levels of performance in bilinguals compared to monolinguals for some tasks, and a different distribution of activation across the two groups (Moreno et al., 2010).

**Table 1** presents methods and outcomes of acceptability judgment experiments in adult, bilingual populations.^2^ Evidently, bilinguals perform similarly to monolinguals only in some tasks and may differ in on-line responses. Bilingualism and variation in the input can affect speakers’ performance in evaluating the structures that (do not) form part of their repertoire. Observing such differences between monolingual and bilingual speakers, the question that arises in this context is whether the greater degree of fluidity that bilectalism, non-standardization, and linguistic proximity entail leads to a performance that is different from that of monolingual and/or bilingual speakers. To this date, no study has compared the performance of monolinguals, bilinguals, and bilectals in a task that measures the ability to identify variants that belong (or not) to their native variety or varieties.

**Table 1:** Relevant studies with adult populations.

Study	Languages	Method	Outcome
Foursha et al. (2006)	English–Spanish	Participants were asked to press one key if they thought the sentence they heard was grammatical and another key, if they thought the sentence was ungrammatical.	(1) Monolinguals outperformed bilinguals on converging over conflicting sentences for very few sentence pairs.
			(2) Bilinguals produced slower overall reaction times.
			(3) Bilinguals and monolinguals did not present different patterns of performance for the conflicting and converging sentences.
Jacobson and Cairns (2008)	English–Spanish	Participants were asked to report acceptability on a binary scale after being orally presented with a sentence.	(1) Monolinguals overwhelmingly rejected hearing or using over-regularizations of the sort *caught* vs. *catched*.
			(2) Bilinguals reported hearing and using some of the over-regularizations to a greater degree than monolinguals.
Moreno et al. (2010)	English–French/Hebrew/Romanian/Russian	Participants were presented with the sentences word-by-word and were asked to press one of the two buttons, answering “yes” if the sentence was good and “no” if there was something wrong with the sentence. The *acceptability* task asked participants to identify errors in grammar or meaning. The *grammaticality* task participants indicated only errors in grammar. ERPs were recorded.	(1) In the acceptability task, bilinguals were less accurate than monolinguals.
			(2) In the grammaticality task, the two groups showed a comparable level of accuracy.
			(3) Bilinguals generated smaller P600 amplitude and a more bilateral distribution of activation than monolinguals.


A recent study investigating the linguistic profile of bilectal speakers of Cypriot Greek (CG) and Standard Modern Greek (SMG) in a written variety-judgment task that superimposed dialectal elements from CG on SMG stimuli revealed important differences between the two groups of speakers across all levels of linguistic analysis (Leivada et al., forthcoming). Despite the fact that this study provided a novel comparison between monolingual and bilectal speakers of two different varieties of Greek, it cannot answer the question of differential performance across monolinguals, bilinguals, and bilectals for two reasons. First, the study tested only school teachers, not the general population. The reasoning behind this was sociolinguistic in nature.

Specifically, Cyprus involves a state of diglossia, with SMG being the sociolinguistically ‘H(igh)’- and CG the ‘L(ow)’-variety. SMG, the official language of the state, is the language used in education and other formal settings.^3^ Pavlou and Papapavlou’s (2004) investigation of dialect use in education puts emphasis on the fact that the language of instruction in Cyprus is SMG and the majority of the textbooks are produced in Greece. As a result, the official policy “forces teachers to adopt as part of their teaching methodology the following principles: SMG should be the exclusive code of instruction and of general use in class; and students should be “corrected” when *using dialect words*, when pronouncing words with a *Cypriot accent*, and when using phonological rules that are part of the *phonological system of GC*” (p. 250; emphasis added).

Despite what the official policy requires, numerous studies have revealed interference of CG in oral and/or written discourse from the students’ perspectives (Pavlou and Christodoulou, 2001; Ioannidou, 2002; Papapavlou and Yiakoumetti, 2003). Experiments focusing on teachers’ output and possible CG interference in *their* repertoire are scarce (Karyolemou, 2006). This is the topic of Leivada et al. (forthcoming). The underlying assumption is that teachers are in a better position than anyone else in Cyprus to demonstrate advanced linguistic performance in the standard language—SMG. In fact, their command of the language should be comparable to that of Hellenic Greeks, monolingual native speakers of SMG, given that they are educated and professionally trained to teach in this variety. The results of Leivada et al. (forthcoming) revealed differences in the performance of bilectal, Greek Cypriot teachers compared to monolingual, Hellenic Greek teachers, as the former were significantly less accurate than the latter in identifying dialectal elements and correctly classifying the test stimuli as SMG or not.

The second reason that Leivada et al. (forthcoming) cannot address the issue of differential performance across monolinguals, bilinguals, and bilectals is that no bilingual group was tested in that study, hence no comparison of bilinguals with bilectals is possible. The present experiment aims to fill this gap in the literature, through investigating potential differences in the performance of monolingual, bilingual, and bilectal populations in a written task and identifying the factors that affect this performance.

### Aims and Predictions

The present study aims to answer the question of whether the differential performance across monolinguals and bilectals reported in Leivada et al. (forthcoming) is the result of being exposed to more than one linguistic system in the course of development. If so, the prediction is that bilectals will perform like bilinguals, since both have exposure to more than one linguistic system. However, if factors inherent to the dialect design such as non-standardization and linguistic proximity between the two varieties come into play, one expects bilectals to perform differently than bilinguals and monolinguals. In sum, the starting point for investigating speakers’ perception of native grammatical variants, can be summarized in the following two possible causes of differential performance across groups.

(1) Non-standardization of CG and its close linguistic proximity to SMG blur the boundaries of grammatical variants and increase grammatical fluidity. Consequently, Greek Cypriots’ perception of their linguistic repertoire is affected by such factors. They become less accurate in spotting the dialectal elements they are presented with in variety judgment task and classifying the test stimuli as CG or SMG.
(2) The differential performance is not due to the existence of two closely related varieties but to the fact that Greek Cypriots are competent in the two varieties. Put differently, their performance is related to the *existence* of two varieties and not to which (and how close or standardized) these two varieties are. As mentioned already, the relevant literature reports that bilinguals perform differently than monolinguals in some acceptability judgment tasks, and this difference could plausibly extend to bilectals.

In sum, bilectals have exposure to both varieties so, in principle, one could think they are in a more privileged position to correctly identify the test stimuli compared to the other groups. We do not favor this possibility, but we acknowledge it as valid hypothesis among others. The aim is to empirically (dis)confirm this idea, especially since it *could* be the case that exposure to more than one language leaves its imprint in the same way across bilingual and bilectal populations. To the best of our knowledge, no other study has compared monolinguals, bilectals, and different type of bilinguals, hence no other study has already ruled out this possibility. If this privilege is not reflected in the performance of bilectals, this could mean that other factors intervene and cloud their ability to identify the grammatical variants that are part of their linguistic repertoires. In this case, the performance of the bilingual group will be the control that can elucidate whether these other factors boil down to (1) or (2).

### Participants

A total of 361 participants took part in this study, divided into four groups:

(i) Hundred Hellenic Greek monolingual speakers of SMG who have acquired language in the monolingual environment of Greece (Group I_HG;_ see **Table 2** for further breakdown);
(ii) Hundred Greek Cypriot bilectals who have been exposed to both SMG and CG in the bilectal context of Cyprus (Group II_GC_);
(iii) Sixty-one Greek Cypriot bilectals who have acquired SMG and CG in the bilectal environment of Cyprus *and* have lived in Greece for more than 1 year during adulthood, most of them studying toward a 4-year university degree (Group III_GC-GR,_ mean age in Greece: 3.6 years); and
(iv) Hundred bilingual speakers of SMG and a second language (Group IV_BI_; participants’ second language is shown in **Table 3**).

**Table 2:** All groups.

Groups		*N*	Age range (in years)	Mean age (in years) (*SD*)
Group I_HG_		100 (77 females)	19–67	32.12 (10.2)
Group II_GC_		100 (49 females)	18–72	32.16 (12.9)
Group III_GC-GR_		61 (35 females)	19–65	33.82 (11.57)
Group IV_BI_	Simultaneous bilinguals	33 (23 females)	20–59	36.6 (10.09)
	Heritage speakers	25 (19 females)	22–56	38.56 (8.51)
	L1 attriters	42 (30 females)	26–80	44.45 (12.03)
	Overall	100 (71 females)	20–80	40.39 (11.09)


*SD stands for Standard Deviation.*

**Table 3:** Languages and types of bilinguals.

Languages	L1 Attriters	Simultaneous bilinguals	Heritage speakers	Total
Albanian		4		4
Bulgarian		1	1	2
Danish	5	2	1	8
English	3	6	3	12
French	1	2	2	5
German	4	7	6	17
German and Norwegian		1		1
Hungarian	2			2
Norwegian	12	3	1	16
Romanian		1		1
Russian		2		2
Spanish	1			1
Swedish	14	3	11	28
Turkish		1		1
Total	42	33	25	100


Group IV_BI_ is further divided in three subgroups:

(i) Thirty-three simultaneous bilinguals that were exposed to both their languages from birth and grew up mainly in Greece;
(ii) Twenty-five heritage speakers that were educated exclusively abroad and learned SMG mainly through the home environment; and
(iii) Fourty-two L1 attriters who grew up in Greece as monolingual speakers of SMG and got exposed to their second language only as adults.

Following recent research (Kaltsa et al., 2015), participants in this last subgroup have spent at least 7 years abroad at the time of testing in order to ensure adequate exposure to the other language. **Table 3** presents the type distribution of bilinguals within Group IV_BI_ and shows the demographics of participants in this group.

All participants were literate adults that had completed secondary education in (mostly public) mainstream schools and were asked to report whether they had a history of neurological or behavioral problems as well as whether they received any speech-pathology treatment. Exclusion criteria included absence of normal articulation, hearing, and (corrected-to-) normal vision, neurological or behavioral problems, and language delay, based on participants’ self-report. Participants that reported receiving speech-pathology treatment, a history of neurological and/or behavioral problems and use of hearing aid were excluded from the analyzed results. All bilingual participants have stated that one of their native languages is SMG. Bilectal participants were born and educated in Cyprus, but due to sociolinguistic reasons, they varied in stating that their native language is CG, SMG, both, or simply Greek. All participants from Group I_HG_ and Group IV_BI_ were tested through an online platform (LimeSurvey^4^), while some participants of Group II_GC_ and Group III_GC-GR_ were tested in our lab.

An important, final note is necessary with respect to the linguistic identity of the bilingual participants. This study is about the perception of *native* grammatical variants. Rothman and Treffers-Daller (2014) argued that monolingualism and nativeness are often used synonymously in an exclusive way. We take their lead in assuming that bilingual speakers, including heritage language learners, are native speakers too. According to Rothman (2009: p. 156), “a language qualifies as a heritage language if it is a language spoken at home or otherwise readily available to young children, and crucially this language is not a dominant language of the larger (national) society.” In the context of the present study, heritage speakers are of interest because it has been argued that their performance may differ from that of non-heritage speakers of the same language with respect to the amount of variation attested (e.g., Lohndal and Westergaard, 2016; see also Montrul, 2002, 2008). All in all, we consider all our speakers, monolinguals, bilinguals, and bilectals, as native speakers of their respective language(s), based on their self-report.

## Materials and Methods

This study employed the written variety-judgment task used in Leivada et al. (forthcoming). This task contains a total of 45 sentences, 30 of which involve the presence of morphemes, syntactic structures, graphemes corresponding to phonological variants, or lexical items that are CG-specific. Each of the 30 sentences includes only one dialectal element. Fifteen sentences function as fillers; these are acceptable sentences of SMG with no dialectal element present. In order to exclude random performance in any linguistic area or condition, each area of testing (syntax, morphology, semantics, phonology, and the lexicon) involves two conditions (e.g., two types of morphemes, graphemes, etc.), and each condition has three items of the attested variant (see **Table 4**). All four core levels of linguistic analysis are examined, plus the lexicon, by the same number of test sentences for each (*n* = 6). All groups of test sentences were randomized across conditions.

**Table 4:** List of areas and conditions tested.

Area [six items per area]	Condition [three items per condition]
Syntax	Clitics
	Case
Morphology	Diminutive suffix –*ui*
	Aspect
Semantics	Nouns
	Verbs
Lexicon	Nouns
	Verbs
Phonology	t ʃ / ʃ / dʒ
	k*^h^*/t*^h^*/p*^h^*


The five areas and their conditions are the following:

(A) Syntax was tested by (i) clitic placement that is licit in CG but not in SMG (i.e., CG enclisis in a declarative clause in indicative mood vs. SMG proclisis in the same syntactic environment) and (ii) the realization of syntactic case that is licit in CG but not in SMG (e.g., *opos* ‘like’ + accusative in CG vs. *opos* ‘like’ + nominative in SMG).
(B) Morphology was investigated through (i) the CG-specific diminutive suffix –*ui* and (ii) the CG marking of grammatical aspect, which in some cases (one of which is the verb *katalavo* ‘understand’, used in the experiment) appears in a morphological combination that is unavailable in SMG, namely, perfective aspect with present tense; in SMG, present tense can only take the morphological suffix of imperfectivity.
(C) Semantics was examined by (i) the use of nouns (*n* = 3) that exist in both SMG and CG, but with a different meaning across varieties (e.g., *pis:a* means ‘chewing gum’ in CG, but ‘tar’ in SMG; in a context where only the former meaning is allowed, the use of this word should be marked as dialectal); and (ii) the use of verbs (*n* = 3) that have different meanings or different thematic requirements across the two varieties.
(D) Phonology was assessed through nouns that involve a phoneme that is CG-specific (e.g., aspirated stops). Apart from the phoneme in question, these nouns are used identically in both varieties. Given that CG lacks an official writing system, the items were written as they appear in dictionaries and thesauri.
(E) Lexicon was tested through the use of CG-specific lexical items: verbs (*n* = 3) and nouns (*n* = 3).

Participants completing the task online were presented with each sentence in written form and were asked to read through each sentence carefully and to classify it as either SMG or CG/dialect (Group II_GC_ and Group III_GC-GR_ were given the former option and all the other groups the latter). Sentences were presented in lower case letters, one at a time, and the software did not allow participants to go back and change their answer. Also, they did not have the option to skip a question. Instructions, written in SMG, were displayed at the top of the window at all times.

Those participants that completed the task in our lab were given a list of sentences of the same format in the form of a booklet, with material presented as in the online task. They were given the same instructions and were ‘supervised’ by a researcher in order to avoid any self-corrections. All participants had no information with respect to how many sentences involved dialectal elements or how many dialectal parts were present per sentence. It was explained to all participants that the presence of even a single dialectal element sufficed to render the classification of a sentence as CG/dialect. Participants had no time limits. Overall, the task took no more than 20 min to complete. This study was carried out in accordance with the recommendations of the Cyprus National Bioethics Committee, with written informed consent from all subjects.

## Results

A dataset of 16,245 sentences was analyzed. We measured four types of responses:

(i) *Correct responses to test items* — identifying a test item as having a dialectal element and correctly classifying the test item as CG/dialect.
(ii) *Wrong responses to test items* — identifying no dialectal element in a test item that has one and failing to classify the test item as CG/dialect.
(iii) *Correct responses to fillers* — identifying no dialectal element in a filler and correctly classifying the test item as SMG.
(iv) *Over-corrections* — identifying a filler as having a dialectal element when there is none and failing to classify the test item as SMG.

**FIGURE 1:**
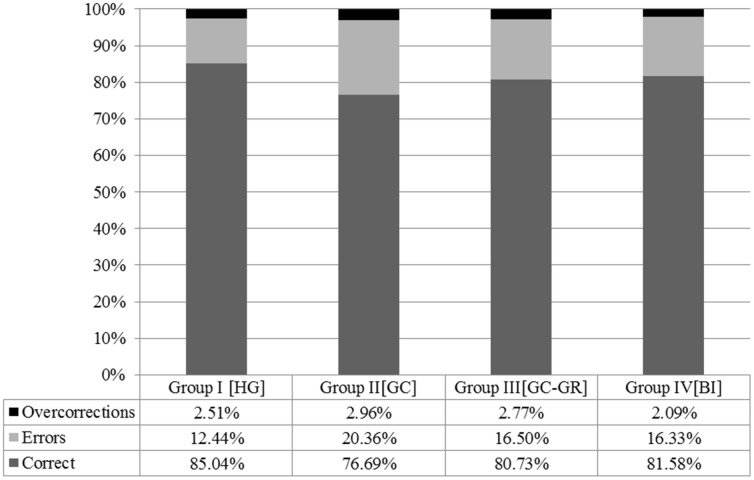
**Overall performance across groups**.

**Figure 1** presents the overall performance across groups. Correct responses for both test items and fillers are grouped together. Wrong responses in test items are presented as ‘errors’ and over-corrections are shown separately. According to a univariate ANOVA test, all types of bilinguals performed similarly in terms of overall errors [*F*_(1,97)_ = 0.162, *p* = 0.85], hence they are grouped together in the presentation of the results.

**Figure 1** shows that Group I_HG_ (the monolinguals) performed significantly better than Group II_GC_ (the bilectals that had not lived in Greece). A univariate ANOVA test showed that there are statistically significant differences between the four groups presented in **Table 2** [*F*_(3,357)_ = 23.61, *p* < 0.05]. A *post hoc* Tukey analysis showed that the differences in terms of errors, including over-corrections, are statistically significant across all groups, with the exception of the difference between Group III_GC-GR_ and Group IV_BI_ (*t* = 0.74, Pr = 0.88).

**FIGURE 2:**
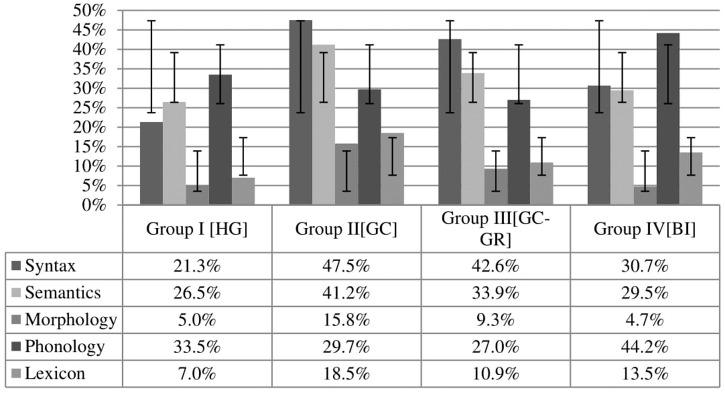
**Errors across groups in the five areas of testing**.

Errors were then analyzed across all conditions. **Figure 2** shows overall percentage of errors in each area of testing: syntax, semantics, morphology, phonology and the lexicon. Fillers are not presented in **Figure 2**; they are analyzed separately below.

### Syntax

Syntax proved particularly difficult for the Groups II_GC_ and III_GC-GR_, with the former performing almost at chance level. As **Figure 2** shows, both bilectal groups performed double the amount of errors of their monolingual peers. Bilinguals (Group IV_BI_) performed worse than monolinguals (Group I_HG_), but considerably better than bilectals (Group II_GC_); the differences are statistically significant in both cases according to a Tukey analysis [(*t* = -3.21, Pr = 0.007) and (*t* = 5.78, Pr < 0.001) respectively].

### Semantics

Semantics is another domain where Group II_GC_ stands out as the only group with errors above 40%. The other group of bilectals performed better (Group III_GC-GR_), but the differences are not so pronounced in semantics as were in the case of syntax. According to a Tukey analysis, the differences that reach statistical significance are found between the bilectals of Group II_GC_ and both bilinguals (*t* = 4.31, Pr < 0.001) and monolinguals (*t* = –5.41, Pr < 0.001).

### Morphology

Compared to the syntactic and semantic conditions, participants did better in morphology. Once more, the highest error rate was at 15.8% for Group II_GC_ with all other error rates below 10%. The only differences that are statistically significant are between the bilectals of Group II_GC_ and all other groups (Group II_GC_-Group I_HG_: *t* = -5.12, Pr < 0.001; Group II_GC_-Group III_GC-GR_: *t* = 2.69, Pr = 0.03; Group II_GC_-Group IV_BI_: *t* = 5.28, Pr < 0.001). For the first time, the group that performed best is not that of monolinguals; bilinguals have an even lower percentage of errors, although the difference between the two is not statistically significant based on a Tukey analysis (*t* = 0.15, Pr = 0.99).

### Lexicon

Concerning the lexical condition, Group II_GC_ has again the highest percentage of errors at 18.5%, followed by the bilinguals (Group IV_BI_). The following differences across groups are statistically significant according to a Tukey analysis: Group I_HG_-Group II_GC_ (*t* = –5.61, Pr < 0.001), Group II_GC_-Group III_GC-GR_ (*t* = 3.21, Pr = 0.007), and Group I_HG_-Group IV_BI_ (*t* = –3.17, Pr = 0.008).

### Phonology

**Figure 2** shows that there is a clear pattern of performance across groups in all conditions apart from phonology, where both monolinguals and bilinguals performed worse than bilectals. Statistically significant differences are found when comparing the bilinguals with any of the other three groups (Group IV_BI_-Group I_HG_: *t* = -3.93, Pr < 0.001; Group IV_BI_-Group II_GC_: *t* = -5.34, Pr < 0.001; Group IV_BI_-Group III_GC-GR_: *t* = -5.49, Pr < 0.001).

The reason for this performance has to do with the task at hand: Phonology was tested through orthography, which may not be the ideal vehicle for testing this domain. As noted in Leivada et al. (forthcoming), the presence of aspirated stops in the task—which are frequently used in CG but do not exist in SMG—is represented in written form through a double occurrence of the relevant consonant. For example, the word *pit:a* ‘pie’ involves one τ ‘t’ in SMG, but two in CG. Given that in previous forms of Greek, this word was spelled with ττ, one can hypothesize that participants of the monolingual group might be familiar with this form, thus failing to mark the relevant sentence as dialectal. Bilectals, however, are strongly aware of this phonological discrepancy since it is one of the most salient characteristics of CG, hence they mark the relevant test structure as such (Leivada et al., forthcoming). Also, in the present study, some of the bilingual participants from Group IV_BI_ had been educated mainly in another language (see the subgroups in **Table 2**) and may not have been able to identify orthographical mismatches from the correct form.

For this very reason, we re-did the overall error analysis without phonology. Not taking phonology into account, a Tukey analysis shows that error differences across all groups remain statistically significant, with the exception of the difference between Group I_HG_ and Group IV_BI_ (*t* = -2.42, Pr = 0.07). In other words, if phonology is disregarded, *monolinguals and bilinguals pattern together* and behave differently than both groups of bilectals.

### Fillers

**FIGURE 3:**
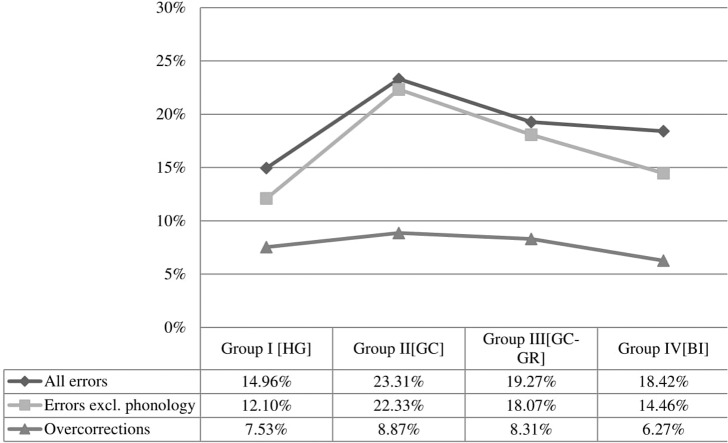
**Overall errors with and without phonology, and over-corrections**.

Fillers reveal an interesting finding of the present study. Recall that fillers are sentences that do not involve any dialectal element. Therefore, mistakes in fillers can be viewed as over-corrections: Participants identify an element as dialectal where there is none. **Figure 3** shows such over-corrections across groups, together with the total percentage of errors with and without phonology.

Running a Tukey analysis of over-corrections across groups,^5^ statistically significant differences are found only between the bilectals of Group II_GC_ and both bilinguals (*t* = 4.94, Pr < 0.001) and monolinguals (*t* = -4.56, Pr < 0.001).

**FIGURE 4:**
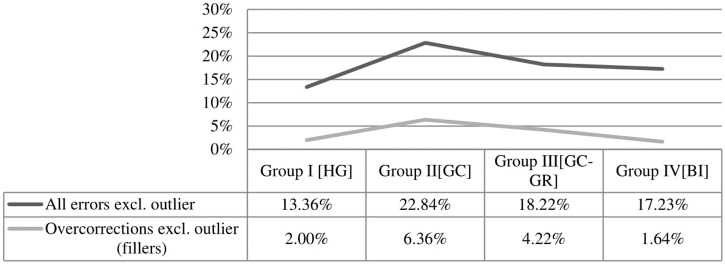
**Overall errors without outlier across all conditions and in fillers**.

**FIGURE 5:**
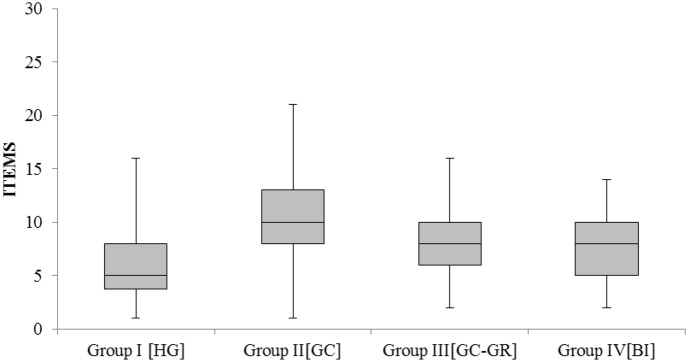
**Dispersion of errors without outlier across all conditions and in fillers**.

A comparative analysis of errors excluding the outlier does not change the aforementioned result that monolinguals and bilinguals pattern together, if phonology is not taken into account. A Tukey analysis confirmed that even if the outlier is excluded, the differences between all groups remain significant apart from the difference between Group I_HG_ and Group IV_BI_ (*t* = -2.203, Pr = 0.12). In other words, the exclusion or inclusion of the outlier does not alter one of the most crucial findings of this experiment: If phonology is disregarded, as it should, monolinguals and bilinguals behave alike. **Figures 4** and **5** show patterns of errors across conditions, excluding the outlier.

An analysis of possible factors affecting Groups’ performance was also performed and revealed that gender and place and/or format of the implementation of the task did not affect the tendencies and results observed above. A univariate ANOVA showed that the bilectals of Group II_GC_ that took the study in the lab (*n* = 39/100) did not perform differently in a statistically significant way from the ones that completed the experiment online [*F*_(1,97)_ = 2.70, *p* = 0.10]. With respect to the bilectals of Group III_GC-GR_, a univariate ANOVA showed that the participants that took the study in the lab (*n* = 11/61) performed differently in a statistically significant way from the ones that completed the experiment online [*F*_(1,59)_ = 5.125, *p* = 0.02], with the latter being better (mean errors 8.28 vs. 10.45 in the lab-based participants). The low number of lab-based participants should be taken into account in interpreting the difference in the performance of these two groups.

## Discussion

The findings of this study suggest that exposure to two different grammars affects speakers’ performance in variety judgment tasks in some levels of linguistic analysis. This result agrees with the differential performance across monolinguals and bilinguals that has been reported in the literature (Jacobson and Cairns, 2008). The relevant literature refers to the performance of bilingual populations, covering *languages* rather than different varieties of the same language. It is less clear what happens when the developmental trajectory features varieties of one language rather than (adequately) different languages.

It has been argued earlier that since bilectals have exposure to both varieties, it is expected that they would perform better than all the other groups in classifying correctly the test stimuli, precisely because they are familiar both with the Standard and the dialectal element. **Figures 3** and **4** suggest that this is not the case; overall, bilectals were less accurate than all the other groups. This could be due to different factors. One possibility is that non-standardization blurs the boundaries of grammatical variants, hence bilectals become less accurate in identifying the variety to which a grammatical variant pertains. Under another scenario, the differential performance of bilectals derives from the fact that these speakers are competent in two linguistic systems. **Figure 3** shows that the group of bilinguals (Group IV_BI_) performed better than both groups of bilectals, and this should not happen if the second scenario was on the right track, because bilinguals have exposure to two linguistic systems too, yet they perform better than bilectals. This entails that the differential performance of bilectals is due to other factors.

For instance, linguistic proximity, defined here as the typological closeness between the varieties one is exposed to, is an important factor that characterizes language development in bilectal settings (Grohmann, 2014; Grohmann and Kambanaros, 2016). Recent research in speakers of closely related varieties has revealed that factors such as non-standardization and close proximity affect speakers’ perception of the variants that exist in their native repertoires in a way that is absent from the performance of their monolingual peers (Leivada et al., forthcoming). The present study is the first to tackle the question of whether this differential performance is the result of being exposed to more than one linguistic system in the course of development or of factors inherent to the dialect design (e.g., lack of standardization, unclear boundaries between variants, linguistic proximity between the varieties, awareness of the fact that some of the structures of the L-variety might be considered incorrect by speakers of the standard, etc.). Pursuing this novel comparison between monolinguals, bilinguals and bilectals, we uncovered significant differences between bilectal and bilingual speakers, providing support for the second scenario: *Bilinguals performed considerably better than bilectals in identifying correctly dialectal elements.*

We aimed to ascertain whether this happens because of the dialect design alone or also because what counts as standard in Cyprus may not always correspond to SMG as spoken in Greece by Hellenic Greeks but to another form of standard (perhaps Cypriot Standard Greek; Arvaniti, 2010). For this purpose, we included a group of participants that grew up as bilectals but spent some time in Greece (>12 months) as adults: Group III_GC-GR_. Results suggest that in the overall calculation of errors, with and without the outlier, Group III_GC-GR_ performs more like the bilinguals of Group IV_BI_ and less like their bilectal peers that had not lived in Greece (Group II_GC_). At the same time, the bilectals of Group III_GC-GR_ were less accurate than the bilinguals of Group IV_BI_, as shown in **Figure 4**. The difference is also evidenced in over-corrections, which can be a marker of linguistic insecurity: As **Figure 4** shows, Group III_GC-GR_ performed more than twice the errors of the bilinguals in fillers. The conclusion to be drawn is that exposure to the standard in Cyprus may not always amount to SMG, which is why prolonged exposure to SMG in Greece makes bilectals behave more like true bilinguals. At the same time, factors inherent to the dialect design have influenced the linguistic development of bilectals: Their performance remains less accurate than that of bilinguals, and an increased degree of linguistic insecurity is still manifested in their performance even *after* prolonged exposure to SMG.

Errors in fillers are interesting because they do not amount to missing a dialectal element superimposed on an otherwise standard form, but to identifying a dialectal element where a dialectal element is not present. The attested higher degree of over-corrections in the two bilectal groups cannot be the result of having two grammars. Bilinguals have two grammars too, yet their performance is comparable to that of monolinguals. Therefore, another explanation should be found regarding the higher degree of over-corrections in bilectals. A possible reason is the linguistic insecurity that often characterizes dialect speakers (Toribio, 2000). Bilectal speakers are eager to show that they are competent in the H-variety, and this may be the cause of the higher degree of over-corrections in their performance. This finding is in perfect agreement with data from child language that come from the bilectal context of Cyprus. As Grohmann and Leivada (2012) have argued, bilectal children’s process of building a sociolinguistic repertoire primarily involves the need to resolve linguistic anxiety and adjust to the H-variety. It is likely that this factor of dialect design drives linguistic performance of bilectal speakers even well past the acquisition period (the ‘socio-syntax of development hypothesis’). This is true also of the group of bilectals that have lived in Greece (Group III_CG-GR_). Despite their prolonged exposure to SMG, they show a higher degree of over-corrections compared to their monolingual and bilingual peers.

A last result that is worth highlighting relates to the performance of the three subgroups within Group IV_BI_. Their performance was found to be so similar that they were grouped together in all calculations mentioned above. This finding agrees with the results of Kaltsa et al. (2015) who examined monolingual speakers of SMG and two types of bilingual speakers (heritage speakers and L1 attriters) of SMG and Swedish in a sentence-picture matching decision task. They found differences in anaphora resolution of overt and null subject pronouns between monolinguals and bilinguals, but *not* between the two groups of bilinguals. The only difference between the two bilingual groups was found in reaction times, with heritage speakers being faster than L1 attriters. The absence of a difference in the off-line measure led Kaltsa et al. (2015, p. 266) to conclude that their results “do not support an age of onset or differential input effects on bilingual performance in pronoun resolution.” Our results seem to fully support this conclusion too across different grammatical conditions and domains of linguistic analysis.

## Conclusion

The results of the present study have confirmed the findings of previous research showing that bilinguals perform differently than monolinguals in acceptability and variety judgment tasks *only* in some linguistic domains. In the written variety-judgment task employed here, if phonology is not taken into account, the differences between monolinguals and bilinguals are not statistically significant. In addition, it was found that bilectals performed worse than both bilinguals and monolinguals, and that the defining characteristics of bilectalism and the dialect design (e.g., linguistic proximity and grammatical fluidity) affect speakers’ performance.

The notion of linguistic proximity is important for the interpretation of the results of the bilectals in the following way. Even though they were the ones that had exposure to both varieties, thus possibly being in a more privileged position to correctly identify the test stimuli compared to the monolinguals, they turned out to be less accurate than the monolinguals. Proximity plays a role in that it facilitates the emergence of mesolectal varieties that blur the limits of different lects. This in turn makes the bilectals less accurate in distinguishing the lect to which each grammatical variant pertains.

This study has shown that the linguistic insecurity that is often found in bilectal speech communities (Toribio, 2000) persists in the form of over-corrections even after prolonged exposure to the H-variety. Last, our comparison of three groups of bilingual speakers did not show significant differences between them, granting new support to the argument of Kaltsa et al. (2015) that age of onset and differential input do not affect performance in off-line measures.

## Ethics Statement

According to the local legislation in Cyprus, ethical approval was not required for this type of study. The study was, however, conducted in accordance with the declaration of Helsinki and written informed consent was obtained from each participant.

## Author Contributions

EL designed the experiment in consultation with EP, MK, and KKG; EL and EP recruited the participants; EL and EP analyzed the results; EL, EP, MK, and KKG wrote up the paper.

## Conflict of Interest Statement

The authors declare that the research was conducted in the absence of any commercial or financial relationships that could be construed as a potential conflict of interest.

## Footnotes

1 Throughout this work, the term ‘bilingualism’ denotes competence in two different languages, whereas the term ‘bilectalism’ is employed when there is great structural proximity between the two varieties of a speaker. We also prefer use of the term ‘bilectal’ over ‘bidialectal’ for purposes of precision: The H-variety in many diglossic speech communities is a standard language, which is a superposed variety and not a dialect.

2 We use the term ‘acceptability’ rather than ‘grammaticality,’ although many studies employ both, sometimes even interchangeably or as if there was a measurable contrast (see **Table 1**). There simply is no list of linguistic stimuli that are grammatical *in and of themselves*, hence we talk about *acceptability*. For further discussion, see also Bard et al. (1996), Schütze (1996), Keller (2000), or Sprouse et al. (2013) and the broader issues laid out in Sprouse and Almeida (2013).

3 For the linguistic reality of Cyprus, see Rowe and Grohmann (2013, 2014) and for attitudes toward CG in the classroom environment see Sophocleous and Wilks (2010), including relevant references cited there.

4 LimeSurvey is an open-source web application used to develop, publish, and collect responses to on-line and off-line surveys.

5 One filler was excluded from these analyses because it involves the word *riγa* ‘ruler,’ which, despite being listed in SMG dictionaries with this meaning, is not frequently used in the language (another word is used instead)—but it is frequently used in CG. As a result, some participants in all groups classified this test sentence as ‘dialectal’(Group I_HG_ at 85%, Group II_GC_ at 44%, Group III_GC-GR_ at 65% and Group IV_BI_ at 71%), whereas in the test design it counts as filler (i.e., non-dialectal/SMG). **Figure 3** includes this filler and **Figures 4** and **5** exclude it.
